# Immune Aging as a Failure of Programmed Cell Death Coordination

**DOI:** 10.3390/ijms27031554

**Published:** 2026-02-05

**Authors:** Hyeong-Min Lee, Eun-Ji Kim, Anamul Hasan, Tae-Bong Kang

**Affiliations:** 1 BK21 Program, Department of Applied Life Science, Graduate School, Konkuk University, Chungju 27478, Republic of Korea; 2 Department of Biotechnology, College of Biomedical & Health Science, Konkuk University, Chungju 27478, Republic of Korea; 3 Research Institute for Biomedical & Health Science (RIBHS), Konkuk University, Chungju 27478, Republic of Korea

**Keywords:** immunosenescence, inflammaging, programmed cell death, apoptosis, necroptosis, pyroptosis

## Abstract

Aging profoundly reshapes the immune system, leading to increased susceptibility to infection, impaired vaccine responses, chronic inflammation, and age-associated inflammatory diseases. While immune aging has traditionally been attributed to defects in immune cell development, signaling, and metabolism, emerging evidence highlights dysregulation of programmed cell death as a central and unifying mechanism. Apoptosis, necroptosis, pyroptosis, and ferroptosis are increasingly recognized not only as terminal cellular events but also as active regulators of immune homeostasis and inflammatory signaling. In aged immune cells, coordination among these death pathways is disrupted, weakening apoptotic resolution and favoring inflammatory forms of cell death that amplify tissue damage and sustain inflammaging. In this review, we summarize current evidence on how aging remodels programmed cell death pathways in the immune system, discuss the molecular mechanisms underlying this network-level shift, and consider potential strategies for restoring immune function by modulating cell death decisions.

## 1. Introduction

Immune aging, or immunosenescence, is characterized by a progressive decline in protective immunity, including impaired pathogen clearance, weakened vaccine responsiveness, and compromised immune memory [1,2,3,4,5]. This functional deterioration is frequently accompanied by inflammaging, a chronic, low-grade inflammatory state implicated in a wide range of age-related diseases such as cardiovascular disorders, neurodegeneration, metabolic syndrome, and cancer [6,7,8,9]. Although these two phenomena are tightly linked, immune aging and inflammaging are conceptually distinct. Inflammaging describes a systemic inflammatory condition, whereas immune aging refers to intrinsic, cell-autonomous and population-level changes within the immune system that precede, shape, and sustain this inflammatory milieu [10].

Most studies on immune aging have focused on altered immune cell composition, stem cell exhaustion, replicative senescence, and defective signaling pathways [11]. In contrast, inflammaging has largely been attributed to persistent innate immune activation, accumulation of senescent cells, and chronic exposure to endogenous danger signals [12,13,14,15,16]. However, the mechanistic bridge connecting immune-intrinsic aging processes to sustained inflammation remains incompletely understood.

Programmed cell death provides a critical conceptual and mechanistic link between immune aging and inflammaging. Under homeostatic conditions, apoptosis enables immunologically silent removal of damaged or excess immune cells, thereby limiting inflammation [17,18]. Aging, however, alters cellular stress responses, mitochondrial integrity, redox balance, and proteostasis, reshaping the decision-making landscape of cell death [19,20,21,22]. Rather than simply increasing cell loss, aging fundamentally disrupts the coordinated regulation of programmed cell death, weakening apoptosis-mediated silent resolution and favoring inflammatory death pathways such as necroptosis, pyroptosis, and ferroptosis. This network-level rewiring transforms immune cell turnover into a persistent source of inflammation, positioning immune aging as a causal driver of inflammaging rather than a passive consequence [23,24,25,26].

In this review, we discuss immune aging through the framework of programmed cell death, highlighting how age-driven changes in death pathway selection within immune cells provide a mechanistic basis for inflammaging.

## 2. Hallmarks of Immune Aging Relevant to Cell Death

Several hallmarks of immune aging directly predispose immune cells to altered death decisions. These changes operate across multiple levels, ranging from stem cell dysfunction to cellular metabolic stress and tissue-level inflammatory cues, collectively reshaping the threshold and modality of programmed cell death.

At the level of hematopoiesis, aging is associated with profound alterations in hematopoietic stem cell (HSC) function. Aged HSCs exhibit clonal skewing, reduced self-renewal capacity, and a bias toward myeloid differentiation, resulting in immune cell populations that are functionally compromised and prone to stress [27,28,29]. Importantly, these progenitor-derived immune cells inherit mitochondrial dysfunction, impaired stress responses, and epigenetic alterations that influence survival and death signaling [27,28,29,30,31,32,33,34]. Such intrinsic defects prime aged immune cells to respond aberrantly to inflammatory or metabolic stress, increasing their susceptibility to dysregulated cell death [35].

At the cellular level, mitochondrial dysfunction represents a central hallmark linking immune aging to altered death pathway selection. Aged immune cells accumulate mitochondrial DNA damage, exhibit reduced oxidative phosphorylation efficiency and generate excessive reactive oxygen species (ROS) [36,37,38]. These changes not only sensitize cells to death-inducing stimuli but also impair the execution of canonical apoptosis by disrupting mitochondrial outer membrane permeabilization and caspase activation [36,39]. Elevated ROS further promotes lipid peroxidation, iron dysregulation, and inflammasome activation, thereby favoring inflammatory forms of cell death such as ferroptosis and pyroptosis [40,41,42,43,44].

Impaired proteostasis and defective autophagy further exacerbate the death pathway imbalance in aged immune cells. Autophagy plays a critical role in maintaining mitochondrial quality control and limiting inflammasome activation [45,46]. With aging, reduced autophagic flux allows damaged mitochondria and protein aggregates to accumulate, amplifying cytosolic danger signals [47]. This environment lowers the threshold for inflammasome activation and necroptotic signaling, while simultaneously limiting the cell’s ability to resolve stress through adaptive survival pathways [48].

The aging immune system is also affected by chronic exposure to inflammatory cytokines, particularly TNF, IL-1β, and type I interferons. Persistent cytokine signaling enforces a state of sublethal stress that continuously engages death-related signaling pathways. In young immune cells, such signals are typically resolved through apoptosis followed by efficient clearance. In aged cells, however, chronic cytokine exposure intersects with reduced caspase-8 activity, altered NF-κB signaling, and metabolic insufficiency, conditions that favor diversion toward necroptosis or pyroptosis rather than apoptotic resolution [12,49].

Beyond cell-intrinsic defects, aging profoundly impairs efferocytosis—the process by which dying cells are recognized and cleared by phagocytes [50]. Aged macrophages and stromal cells exhibit reduced capacity to engulf apoptotic cells, leading to the accumulation of uncleared corpses within tissues [51,52]. These dying cells frequently undergo secondary necrosis, releasing damage-associated molecular patterns (DAMPs) that further activate innate immune receptors and reinforce inflammatory cell death pathways in neighboring cells [14,53]. Thus, inadequate clearance of apoptotic cells shifts apoptosis from a silent process to a chronic inflammatory stimulus [50,54].

**Figure 1:**
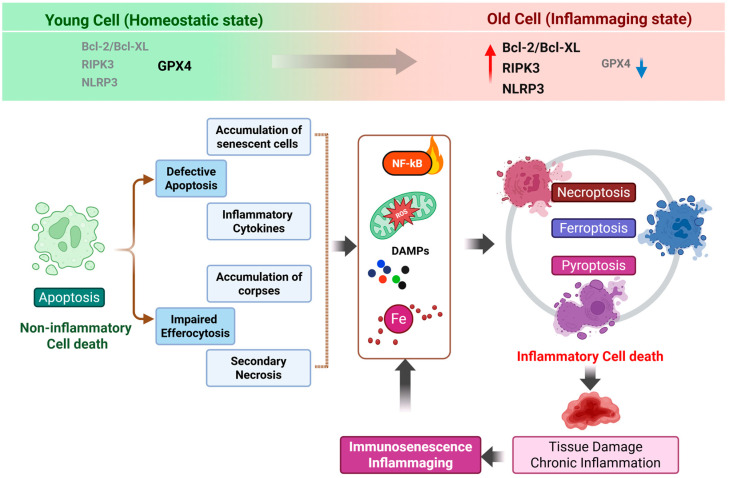
From apoptosis to inflammatory cell death: a vicious cycle underlying immune aging. This schematic illustrates an age-associated shift in cell death programs from homeostatic apoptosis toward inflammatory forms of regulated cell death, including necroptosis, pyroptosis, and ferroptosis. Comparative expression patterns of key regulatory molecules in cells from young versus aged conditions are indicated at the top, highlighting increased Bcl-2/Bcl-XL, RIPK3 and NLRP3 expression (the red upward arrow) and reduced GPX4 levels in aged cells (the blue downward arrow). During aging, reduced efficiency of apoptosis and a decline in efferocytosis lead to the accumulation of dysfunctional cells and cellular debris within tissues. These uncleared apoptotic cells progressively undergo secondary necrosis, resulting in the release of damage-associated molecular patterns (DAMPs) and pro-inflammatory cytokines. The persistent presence of these signals sustains NF-κB activation and reinforces a chronic inflammatory milieu. In parallel, mitochondrial dysfunction–derived reactive oxygen species (ROS) and iron dysregulation further exacerbate cellular stress, shifting cell fate decisions away from immunologically silent apoptosis toward inflammatory forms of cell death, such as necroptosis, pyroptosis and ferroptosis. The ensuing release of additional inflammatory mediators amplifies tissue damage, accelerates immune cell dysfunction and senescence, and establishes a self-perpetuating vicious cycle that underlies immune aging and inflammaging. Created in BioRender. Kang, T. (2026). https://BioRender.com/ua8m688 (accessed on 2 February 2026).

Together, these hallmarks establish an immune environment in which cell death is not only more frequent but also qualitatively altered. With aging, immune cells transition from tightly regulated, immunologically silent apoptosis toward inflammatory and metabolically driven death that triggers systemic inflammation. This rewiring of death pathway selection provides a mechanistic foundation for understanding how immune aging actively promotes inflammaging rather than merely accompanying it. Consistent with these functional alterations, aging is accompanied by a progressive shift in the expression and activity of key cell death regulators, including increased signaling capacity of necroptotic and inflammasome pathways and diminished antioxidant defenses that normally protect against lipid peroxidation (Figure 1) [55,56,57,58,59].

## 3. Apoptosis in Immune Aging

Apoptosis is the dominant homeostatic cell death mechanism in the immune system, ensuring the safe elimination of excess, damaged, or autoreactive cells without affecting tissue integrity and immunological quiescence [60,61,62]. Although intrinsic and extrinsic pathways are tightly regulated, apoptosis maintains immune cell homeostasis, shapes lymphocyte repertoires, and facilitates the resolution of immune responses without triggering inflammation. However, aging profoundly alters the regulation, execution, and resolution of apoptosis, fundamentally changing its functional outcome within the immune system [50,63,64].

Importantly, immune aging does not simply increase or decrease apoptotic frequency in a uniform manner. Rather, aging introduces context-dependent dysregulation, in which certain immune cell populations become resistant to apoptosis, while others show heightened sensitivity. This imbalance disrupts immune composition and compromises immune competence [3,6,65]. In adaptive immunity, repeated antigen exposure, telomere attrition, epigenetic drift, and metabolic insufficiency collectively drive T cell exhaustion. Exhausted and terminally differentiated memory T cells frequently acquire resistance to apoptosis, allowing them to persist despite functional impairment [66,67,68,69,70]. The accumulation of such dysfunctional memory T cells contributes to reduced immune plasticity and diminished responsiveness to new antigens.

In contrast, naïve T cells in aged individuals often display increased susceptibility to apoptosis [71,72]. Heightened sensitivity to cytokine withdrawal, oxidative stress, and mitochondrial damage leads to accelerated loss of naïve T cells, resulting in contraction of the T cell receptor repertoire. This selective depletion compromises adaptive immune diversity and represents a defining characteristic of immunosenescence. Thus, aging reshapes apoptosis in a population-specific manner, promoting the survival of dysfunctional immune cells while eliminating those required for robust immune renewal [73,74,75,76].

In innate immune cells, aging-related changes are characterized by insufficient apoptotic turnover instead of simple resistance to pro-apoptotic signaling. Many studies indicate that aged innate immune cells exhibit impaired activation of some caspases, altered mitochondrial outer membrane permeabilization, and dysregulated death receptor signaling. These defects do not necessarily prevent cell death but instead delay or incompletely execute the apoptotic program. Such incomplete or defective apoptosis leads to a permissive state in which cells remain metabolically active despite being marked for death signals [36,77,78,79,80].

This incomplete apoptotic state carries significant functional implications [17,81,82,83,84]. Under conditions of chronic inflammatory signaling—such as persistent exposure to TNF, IL-1β, or type I interferons—aged immune cells with compromised apoptotic machinery tend to redirect death signaling toward alternative, inflammatory pathways [15,85]. In particular, reduced caspase-8 activity removes a critical checkpoint that normally suppresses necroptosis [86,87,88]. As a result, aging transforms apoptosis from a terminal, anti-inflammatory endpoint into an unstable intermediate that facilitates engagement of necrotic death programs.

In addition to execution defects in apoptosis, aging also profoundly impairs the resolution phase of apoptosis, namely the efficient clearance of apoptotic cells by phagocytes [51]. Efferocytosis is essential for maintaining immune tolerance and preventing secondary inflammation [50]. However, aged macrophages exhibit reduced phagocytic capacity, altered expression of “eat-me” receptors, and diminished responsiveness to apoptotic signals, consequently leading to the prolonged persistence of apoptotic cells in tissues [51].

Persisting apoptotic cells frequently undergo secondary necrosis, leading to plasma membrane rupture and release of damage-associated molecular patterns (DAMPs), including nuclear DNA, HMGB1, and oxidized lipids [54,89,90]. This process converts an otherwise immunologically silent form of cell death into a potent inflammatory stimulus [91]. The accumulation of secondary necrotic cells reinforces innate immune activation, perpetuates cytokine production, and further lowers the threshold for inflammatory cell death in neighboring immune cells [50,54,92,93]. Therefore, defective apoptotic clearance acts as a self-reinforcing driver of chronic inflammation.

**Figure 2:**
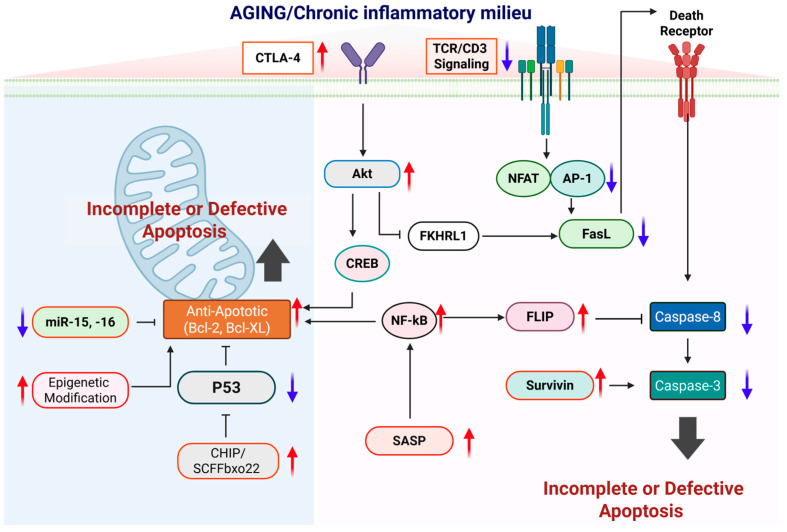
Molecular mechanisms underlying impaired apoptotic control during aging. This schematic depicts how aging and a chronic inflammatory milieu progressively disrupt apoptotic regulation at multiple molecular checkpoints, leading to incomplete or defective apoptosis and the accumulation of apoptosis-resistant cells. In aged immune cells, increased expression of inhibitory receptors such as CTLA-4 and attenuated TCR/CD3 signaling weaken pro-apoptotic transcriptional programs mediated by NFAT and AP-1, resulting in reduced FasL expression and impaired activation of the extrinsic apoptotic pathway. At the same time, pro-survival signaling pathways, including Akt–CREB and persistent NF-κB activation, are enhanced and drive the upregulation of anti-apoptotic molecules such as Bcl-2, Bcl-XL, FLIP, and survivin, while suppressing caspase-8 and caspase-3 activation downstream of death receptor signaling. Within the intrinsic pathway, aging-associated mitochondrial alterations, epigenetic modifications, reduced expression of pro-apoptotic microRNAs (miR-15 and miR-16), and enhanced CHIP/SCFFbx22-mediated p53 degradation or functional suppression further reinforce resistance to apoptosis. In parallel, chronic inflammatory cues and senescence-associated secretory phenotype (SASP) signaling sustain NF-κB–dependent survival programs, thereby stabilizing an anti-apoptotic state. Red upward arrows indicate pathways or factors that are increased with aging, whereas blue downward arrows denote those that are diminished. Created in BioRender. Kang, T. (2026) https://BioRender.com/uk3uq46 (accessed on 2 February 2026).

Collectively, these alterations suggest that apoptosis in the aging immune system is not simply a matter of being diminished or enhanced; rather, it is fundamentally reconfigured and functionally compromised. Aging weakens apoptosis as a protective barrier that normally constrains inflammation and prevents inappropriate activation of necrotic death pathways. Instead, apoptotic dysregulation creates the cellular environment to favor the inflammatory cell death pathway (Figure 2) [25,26,50,94,95].

Thus, apoptosis occupies a pivotal role in immune aging—not only as a victim of age-associated cellular stress, but also as a gatekeeper whose failure enables the emergence of necroptosis, pyroptosis, and other inflammatory death pathways. Understanding how apoptotic integrity is lost with age is therefore essential for deciphering the broader reprogramming of cell death pathways that underlie immune dysfunction and inflammaging [6,95,96,97].

## 4. Necroptosis as an Inflammatory Amplifier in Aging

**Figure 3:**
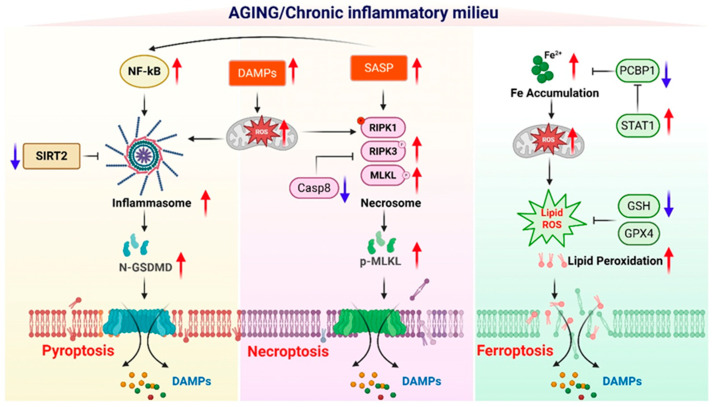
Molecular reprogramming of inflammatory cell death pathways in the aging environment. This schematic illustrates how the aging-associated chronic inflammatory milieu selectively reprograms molecular pathways to promote distinct forms of inflammation-regulated cell death, including pyroptosis, necroptosis, and ferroptosis. During aging, sustained activation of NF-κB signaling, increased accumulation of damage-associated molecular patterns (DAMPs), and enhanced senescence-associated secretory phenotype (SASP) collectively amplify mitochondrial dysfunction and reactive oxygen species (ROS) production, creating a permissive environment for inflammatory cell death. In the pyroptotic pathway, reduced SIRT2 activity and heightened inflammasome activation facilitate caspase-dependent cleavage of gasdermin D (GSDMD), leading to membrane pore formation and the release of DAMPs. In parallel, aging-associated suppression of caspase-8 activity, together with increased RIPK1, RIPK3, and MLKL phosphorylation, favors necrosome assembly and execution of necroptosis via MLKL–mediated membrane disruption. Ferroptosis is promoted by age-related iron accumulation, enhanced Fe^2+^-driven mitochondrial ROS generation, impaired iron chaperoning by PCBP1, and altered STAT1 signaling, which collectively exacerbate lipid peroxidation. Concurrent depletion of antioxidant defenses, including reduced glutathione (GSH) levels and diminished GPX4 activity, further sensitizes cells to ferroptotic death. Red upward arrows indicate molecular pathways or factors that are increased in the aging or chronic inflammatory environment, whereas blue downward arrows denote pathways that are diminished with aging. The release of DAMPs from each inflammatory cell death modality reinforces chronic inflammation, thereby sustaining a feed-forward loop that exacerbates tissue dysfunction and immune aging. Created in BioRender. Kang, T. (2026) https://BioRender.com/3i5cyox (accessed on 2 February 2026).

Necroptosis is a regulated form of necrotic cell death mediated by the RIPK1–RIPK3–MLKL signaling axis [88,98,99]. Under physiological conditions, necroptosis functions as a protective host defense mechanism, eliminating infected cells when pathogens actively suppress apoptotic machinery [100,101,102]. However, accumulating evidence suggests that aging creates a cellular and tissue environment in which necroptosis is aberrantly activated, even in the absence of infection [82,103,104]. Rather than serving as a backup defense pathway, necroptosis increasingly operates as a maladaptive death program that amplifies inflammation in aged tissues [58,105] (Figure 3).

Multiple age-associated factors converge to lower the threshold for necroptotic activation in immune cells [103]. Reduced caspase-8 expression or activity, a hallmark of aging immune cells, removes a critical inhibitory checkpoint that normally suppresses RIPK3 activation [86,106]. At the same time, chronic exposure to inflammatory cytokines—particularly TNF—provides sustained upstream signaling via death receptors [107]. Oxidative stress and mitochondrial dysfunction lower the threshold for RIPK1 activation by undermining the robustness of survival signaling [108,109]. Together, these conditions favor the assembly of the necrosome complex and execution of necroptosis in aged immune cells.

Macrophages and tissue-resident immune cells appear particularly susceptible to necroptotic reprogramming during aging [104]. These cells are chronically exposed to inflammatory cues and metabolic stress within aging tissues and often exhibit impaired apoptotic resolution, as discussed in the previous section. In this context, necroptosis becomes a dominant alternative fate when apoptotic checkpoints fail. Execution of necroptosis leads to MLKL-mediated plasma membrane permeabilization, resulting in rapid cell lysis and uncontrolled release of intracellular contents [110,111,112].

Necroptosis is highly immunogenic, characterized by the extensive release of damage-associated molecular patterns (DAMPs). Key inflammatory mediators such as HMGB1, mitochondrial DNA, ATP, and oxidized lipids are released, driving a pro-inflammatory phenotype [113,114]. These molecules engage pattern recognition receptors such as TLRs and cytosolic DNA sensors in neighboring immune and stromal cells, triggering further production of pro-inflammatory cytokines [115,116,117]. Thus, necroptosis acts as both a result and a driver of inflammatory stress, creating a vicious cycle.

Evidence from aged animal models supports a direct link between necroptosis and chronic inflammation. Increased expression and activation of RIPK3 and MLKL have been observed in aged tissues prone to inflammatory degeneration, including the intestine, liver, brain, and kidney [82,104]. Genetic or pharmacological inhibition of necroptotic signaling in these models often attenuates inflammatory pathology and tissue damage, suggesting that necroptosis is not merely a marker of immune aging but a functional driver of age-associated inflammation [58,104].

Importantly, necroptosis exhibits extensive crosstalk with other programmed cell death pathways in the context of aging. RIPK3 signaling serves as a bridge, triggering inflammasome activation and IL-1β release, which integrates necroptotic and pyroptotic responses [118,119,120]. In aged immune cells, such crosstalk further amplifies inflammatory output and reinforces inflammaging. Thus, necroptosis is not an isolated process but a central hub that connects various inflammatory death programs.

Collectively, these findings position necroptosis as a potent inflammatory amplifier in the aging immune system. Through the induction of membrane rupture and subsequent DAMP release, necroptosis transforms immune aging from passive attrition to active inflammatory progression. Understanding how necroptotic signaling is engaged and regulated in aged immune cells is, therefore, essential for identifying therapeutic strategies aimed at breaking the cycle of inflammaging.

## 5. Pyroptosis and Inflammasome Activation in Immune Aging

Pyroptosis is a highly inflammatory form of programmed cell death driven by inflammasome activation and gasdermin-mediated membrane pore formation [121,122,123]. Unlike apoptosis, pyroptosis is intrinsically pro-inflammatory, coupling cell death with the rapid release of cytokines and intracellular danger signals. Accumulating evidence suggests that aging markedly lowers the threshold for inflammasome activation, rendering aged tissues highly susceptible to pyroptosis-driven immune decline (Figure 3) [124,125,126].

Aging is characterized by an elevated basal priming of the NLRP3 inflammasome [124,127]. At the transcriptional level, aged immune cells often exhibit sustained NF-κB activation due to chronic exposure to inflammatory cytokines and endogenous danger signals [128,129]. This persistent priming state ensures elevated expression of inflammasome components such as NLRP3 and pro–IL-1β, effectively sensitizing aged cells to secondary activation signals [126,130]. Consequently, stimuli that would be insufficient to trigger inflammasome activation in young immune cells can readily induce pyroptotic responses in aged cells [127].

Various hallmarks of cellular aging act synergistically to facilitate inflammasome activation. In particular, mitochondrial dysfunction triggers a pro-inflammatory cascade by releasing reactive oxygen species (ROS) and mitochondrial DNA into the cytoplasm [131,132]. In parallel, age-associated lysosomal instability facilitates leakage of cathepsins, while dysregulated ion homeostasis—particularly potassium efflux—further lowers the activation threshold [133,134,135]. Together, these intracellular stress signals create a permissive environment for the formation of inflammasome assembly and caspase-1 activation.

Macrophages and microglia are particularly vulnerable to pyroptotic reprogramming during aging. As long-lived, tissue-resident immune cells, they gradually accumulate metabolic stress, damaged organelles, and persistent inflammatory cues over time [126,136,137]. In aged macrophages, enhanced inflammasome activation leads to excessive cleavage of gasdermin D, resulting in membrane pore formation and release of IL-1β and IL-18. Similarly, aged microglia display exaggerated inflammasome responses that contribute to neuroinflammation and neurodegenerative pathology [138,139]. Rather than promoting efficient pathogen clearance or tissue repair, repeated or sustained pyroptotic activation drives tissue dysfunction and reinforces chronic inflammation.

Inflammaging associated with senescence primes and sustains inflammasome activity, driving chronic IL-1β/IL-18 signaling. Through positive-feedback loops, this persistent signaling transforms pyroptosis from an acute, self-limiting defense mechanism into a chronic, feed-forward inflammatory cycle. Cytokines released from pyroptotic cells further prime neighboring immune cells, amplify local cytokine networks, and exacerbate tissue damage [124,140,141,142,143]. As a result, this persistent activation state obscures the line separating beneficial immunity from destructive inflammation, encapsulating the essence of inflammaging [126].

Emerging evidence also highlights extensive crosstalk between pyroptosis and other inflammatory cell death pathways in aged immune systems [144,145]. Inflammasome activation can promote necroptotic signaling through RIPK3-dependent mechanisms, while necroptosis-associated DAMP release further enhances inflammasome activation in surrounding cells [120,146,147]. In aged immune cells, where apoptotic checkpoints are weakened, this coordinated activation of pyroptosis and necroptosis amplifies inflammatory output far beyond that produced by either pathway alone [82,148]. Consequently, aging results in an integrated network of inflammatory cell death rather than the activation of discrete, independent pathways.

Collectively, these observations define inflammasome-dependent pyroptosis as a key effector mechanism that integrates immune aging with systemic inflammation. By linking the detection of cellular stress to explosive membrane rupture and cytokine release, pyroptosis transforms age-associated immune dysfunction into persistent inflammatory signaling. Understanding how inflammasome activation is dysregulated with age and how pyroptosis integrates with parallel death pathways is, therefore, essential for deciphering the molecular basis of immune aging and identifying strategies to mitigate inflammaging.

## 6. Ferroptosis and Metabolic Vulnerability of Aged Immune Cells

Ferroptosis is an iron-dependent, lipid peroxidation–driven form of regulated cell death that has recently emerged as a critical contributor to immune dysfunction. Unlike apoptosis or inflammasome-driven cell death, ferroptosis is tightly linked to cellular metabolism, redox balance, and iron homeostasis [59,149]. Aging profoundly alters these metabolic parameters, rendering aged cells increasingly vulnerable to ferroptotic stress (Figure 3) [150,151].

Aged immune cells frequently exhibit increased intracellular iron accumulation, impaired antioxidant capacity, and dysregulated lipid metabolism [152,153,154]. Systemic iron homeostasis becomes less tightly regulated with age, leading to iron deposition in multiple tissues. At the cellular level, reduced expression or activity of antioxidant systems—such as glutathione, GPX4, and NADPH-generating pathways—limits the ability of aged immune cells to detoxify lipid peroxides [59,155]. Concurrently, age-associated changes in lipid composition, including increased polyunsaturated fatty acid content in cellular membranes, further sensitize cells to lipid peroxidation. Together, these alterations create a metabolic landscape that strongly favors ferroptotic cell death [23,151,156].

Macrophages play a pivotal role in this process. As key regulators of iron recycling and storage, they are continuously exposed to iron flux and oxidative stress [157,158]. In aging tissues, macrophages accumulate excess iron and display impaired mitochondrial function and redox control, making them particularly susceptible to ferroptosis [157]. Ferroptotic death of macrophages not only compromises innate immune capacity but also disrupts local iron homeostasis, further amplifying oxidative stress within the tissue microenvironment [158,159,160].

Emerging evidence suggests that ferroptosis also affects adaptive immune cells [161]. T cells depend on strictly controlled redox signaling for activation, proliferation, and survival [162]. In aged T cells, compromised antioxidant defenses and mitochondrial dysfunction increase susceptibility to lipid peroxidation–induced damage [73,75,163]. Ferroptotic loss of T cells may therefore contribute to immune exhaustion, impaired effector function, and reduced immune surveillance in aging organisms [164,165].

Importantly, the impact of ferroptosis extends beyond depletion of immune cell populations, as the resulting oxidized phospholipids and lipid-derived mediators act as potent inflammatory signals [166,167]. These oxidized lipids can activate pattern recognition receptors (PRRs), modulate macrophage polarization, and amplify cytokine production in neighboring immune and stromal cells [168,169,170]. Thus, ferroptosis contributes to inflammaging not only by reducing immune cell viability but also by actively shaping a pro-inflammatory tissue milieu.

Although ferroptosis remains less extensively studied in the context of immune aging than apoptosis, necroptosis, or pyroptosis, accumulating evidence supports its relevance to age-associated inflammation and immune dysfunction. Notably, ferroptosis exhibits mechanistic crosstalk with other cell death pathways through shared upstream drivers, including mitochondrial dysfunction, ROS accumulation, and metabolic insufficiency [167,171,172]. These shared stressors suggest that ferroptosis is integrated into a broader network of inflammatory and metabolic cell death programs that are rewired during aging.

Collectively, ferroptosis shows how metabolic changes contribute to the aging of the immune system. By coupling iron dysregulation, oxidative stress, and lipid peroxidation to immune cell loss and inflammatory signaling, ferroptosis provides a mechanistic link between metabolic aging and chronic inflammation. Further investigation into ferroptotic regulation in immune cells will be essential for fully understanding immune aging and identifying metabolic interventions capable of restoring immune resilience in aged tissues.

## 7. Crosstalk Among Cell Death Pathways in Immune Aging

Programmed cell death pathways do not function as isolated, linear cascades; instead, they form a tightly interconnected regulatory network whose structure and functional balance are profoundly remodeled during aging. In young and healthy immune systems, apoptosis serves as the dominant and protective default, effectively resolving cellular stress and limiting inflammatory signaling [173]. Alternative death programs such as necroptosis, pyroptosis, and ferroptosis are tightly constrained and typically engaged only under specific pathological conditions. Aging disrupts this hierarchy, weakening apoptotic checkpoints and enabling coordinated activation of inflammatory and metabolic cell death pathways.

A master regulator in this network is caspase-8, which functions as a molecular gatekeeper between apoptosis and necroptosis [95,174]. Under homeostatic conditions, caspase-8 activity ensures apoptotic execution while actively suppressing RIPK3-dependent necroptotic signaling. In aged immune cells, however, reduced caspase-8 expression or activity—driven by chronic inflammatory stress, mitochondrial dysfunction, and altered redox balance—destabilizes this checkpoint [25,145]. Consequently, death receptor signaling that would normally lead to apoptosis is shunted toward necroptosis, particularly in macrophages and tissue-resident immune cells exposed to persistent TNF signaling.

Mitochondrial dysfunction serves as another critical hub linking multiple cell death modalities within the context of immune aging [39,175]. Mitochondria integrate metabolic status, redox signaling, and innate immune sensing, placing them at the intersection of apoptosis, pyroptosis, and ferroptosis. In aged immune cells, mitochondrial damage leads to impaired apoptotic signaling, excessive production of reactive oxygen species, and release of mitochondrial DNA into the cytosol [75,176,177]. These events simultaneously promote inflammasome activation and reinforce the sensitivity to oxidative damage. Thus, mitochondrial aging appears to shift cell death decisions toward inflammatory and oxidative outcomes by bridging disparate death programs.

Inflammasome signaling further reinforces this network-level crosstalk. Activation of inflammasomes, particularly NLRP3, not only drives pyroptotic cell death but also intersects with necroptotic signaling [178,179,180]. RIPK3 and MLKL have been shown to modulate inflammasome activation, while necroptosis-associated DAMP release provides potent secondary signals that amplify inflammasome priming in neighboring cells [146,147,181,182]. In aging tissues, where basal inflammasome activity is already heightened [125,126], this bidirectional interaction establishes self-sustaining inflammatory loops that propagate cell death and inflammation across immune populations.

Finally, the metabolic dimension of ferroptosis is integrated into this broader stress–death–inflammation axis. Oxidative stress derived from ferroptotic processes can exacerbate the mitochondrial dysfunction and inflammasome activation mentioned above, while inflammatory cytokines generated by pyroptosis and necroptosis further disrupt cellular redox balance [120,132,183]. In this way, ferroptosis does not operate independently but is integrated into a broader stress–death–inflammation axis characteristic of immune aging.

Viewing immune aging through this network-based framework reframes immunosenescence as a failure of cell death coordination rather than a simple change in cell quantity. Understanding how aging rewires the crosstalk among apoptosis, necroptosis, pyroptosis, and ferroptosis is therefore essential for developing strategies that restore immune balance in aged tissues. Importantly, accumulating evidence from neurodegenerative disorders such as Alzheimer’s disease demonstrates that these death pathways frequently coexist and dynamically switch, providing a disease-relevant framework for the network-based model of immune aging (Table 1).

## 8. Therapeutic Implications

Targeting programmed cell death pathways offers novel and conceptually distinct opportunities to modulate immune aging. Unlike conventional anti-inflammatory approaches that primarily suppress downstream cytokine signaling, interventions aimed at cell death decisions act upstream, at the level of cellular fate determination. By restoring appropriate death pathway selection, such strategies have the potential to rebalance immune homeostasis while preserving essential host defense mechanisms.

Pharmacological inhibition of necroptotic signaling, particularly through targeting RIPK1, represents a promising approach to limit inflammatory cell death in aged tissues [58,195,196]. Preclinical studies in inflammatory and degenerative disease models have demonstrated that RIPK1 inhibition can attenuate tissue damage and chronic inflammation without broadly suppressing immune responsiveness [197,198]. In the context of immune aging, such interventions may prevent the inappropriate engagement of necroptosis that arises from apoptotic checkpoint failure [104,199].

Similarly, suppression of inflammasome activation offers therapeutic potential for mitigating age-associated inflammation [126,200,201,202]. Targeting key components of inflammasome signaling, such as NLRP3 or caspase-1, may reduce excessive pyroptosis and cytokine release in aged innate immune cells. Importantly, selective modulation of inflammasome activity—rather than complete inhibition—may be required to maintain protective immune responses while limiting chronic inflammatory signaling.

Modulation of ferroptosis introduces a complementary metabolic strategy for immune rejuvenation [59,151,203,204]. Enhancing antioxidant capacity, regulating iron homeostasis, or stabilizing lipid metabolism may reduce ferroptotic vulnerability in aged immune cells. Such approaches are particularly attractive in the aging setting, as they address upstream metabolic and redox imbalances that simultaneously influence multiple cell death pathways.

Beyond direct pharmacological interventions, micronutrient supplementation and metabolic modulation represent additional, potentially safer strategies for long-term application in aging populations. Nutrients that support mitochondrial integrity, redox balance, and cellular stress resilience may indirectly reprogram death pathway selection, restoring the dominance of non-inflammatory apoptotic resolution. These interventions may be especially valuable for preventing the gradual escalation of inflammaging rather than treating advanced inflammatory pathology.

Collectively, these therapeutic strategies highlight a shift from symptom-oriented anti-inflammatory treatments toward mechanism-based modulation of immune cell fate. Targeting the core controls of cell death can lower aging-related inflammation while keeping the immune competence. This offers a lasting solution for healthy aging.

Despite the compelling mechanistic rationale, translating cell death–based interventions into clinical practice remains challenging. Programmed cell death pathways are highly context- and cell type–dependent, and their long-term or systemic modulation may compromise host defense, tissue repair, or immune balance in aged individuals. In addition, the lack of reliable biomarkers to monitor death pathway engagement in vivo and the pronounced heterogeneity of immune aging complicate patient stratification and therapeutic optimization. Addressing these limitations will be critical for bridging mechanistic insights with safe and effective clinical applications.

## 9. Future Perspectives

Despite significant progress in understanding how programmed cell death pathways are rewired during immune aging, many fundamental questions remain unresolved.

A key challenge is determining how cell death decisions vary at the single-cell level within aged immune populations. Aging increases cellular heterogeneity, raising the possibility that distinct subpopulations within the same immune lineage may preferentially engage different death pathways in response to identical stimuli. Dissecting this heterogeneity will be essential for understanding why inflammatory cell death becomes dominant in aging tissues.

Tissue context represents another major knowledge gap. Immune cells reside in highly diverse microenvironments, each characterized by distinct metabolic conditions, stromal interactions, and inflammatory cues. How apoptosis, necroptosis, pyroptosis, and ferroptosis are differentially engaged across tissues such as barrier organs, lymphoid sites, and immune-privileged regions remains largely unexplored.

An especially intriguing and clinically relevant question is whether immune aging can be reversed or reprogrammed by resetting cell death thresholds. If immune dysfunction reflects dysregulated death pathway coordination rather than irreversible damage, restoring apoptotic control or limiting inflammatory cell death may partially rejuvenate immune function.

Addressing these issues will require systems-level approaches that integrate single-cell, spatial, and in vivo analyses to move beyond pathway-centric models of cell death in aging immunity.

## 10. Conclusions

Aging transforms immune cell death from a quiet process to a loud, inflammatory one, converting physiological cell turnover into a persistent source of inflammation and tissue damage. By integrating apoptosis, necroptosis, pyroptosis, and ferroptosis into a unified framework, this review proposes that immune aging reflects a breakdown in the coordination of the cell death program rather than a simple accumulation of cell damage. The convergence of mitochondrial dysfunction, redox imbalance, chronic inflammatory signaling, and impaired clearance mechanisms rewires death pathway selection toward inflammatory outcomes, thereby providing a mechanistic foundation for inflammaging.

This perspective also carries important therapeutic implications. Targeting upstream regulatory nodes that govern cell death decisions, rather than suppressing downstream inflammatory mediators alone, may offer a more effective strategy to restore immune homeostasis while preserving host defense. Interventions that stabilize apoptotic checkpoints, limit inappropriate inflammatory cell death, or correct underlying metabolic vulnerabilities may enable partial reprogramming of immune aging.

Ultimately, understanding immune aging will require moving beyond single, linear pathways and instead examining how cell death networks are regulated as an integrated system across different cell types and stages of aging. This broader view suggests that immune aging is not fixed or inevitable, but rather a modifiable state shaped by how cell fate decisions are controlled. Reframing immune aging in this way may open new opportunities to reduce chronic inflammation and preserve healthy immune function throughout life.

## Figures and Tables

**Table 1:** Evidence for the coexistence of apoptosis, necroptosis, pyroptosis, and ferroptosis in Alzheimer’s disease and their switching mechanisms.

Type	Triggers in AD	Key Molecules	Evidence in AD	Switching Mechanism	References
Apoptosis	Amyloid-β accumulation, mitochondrial dysfunction, oxidative stress	Caspase-3, Caspase-9, Bax/Bcl-2	Increased apoptotic neurons in post-mortem AD brains and an AD mouse model	Inhibition or exhaustion of caspase-8 under inflammatory conditions may divert cell fate to necroptosis	[174,184,185]
Necroptosis	TNF-α, Amyloid-β toxicity, chronic neuroinflammation	RIPK1, RIPK3, MLKL	Elevated RIPK3 and MLKL activation in AD patients’ brains and APP/PS1 mice	RIPK3 can promote NLRP3 inflammasome activation, linking necroptosis to pyroptosis	[180,186,187,188,189,190]
Pyroptosis	Microglial inflammasome priming, Amyloid-β fibrils, DAMPs	NLRP3, Caspase-1, GSDMD	Increased NLRP3 activation and IL-1β release in AD patients’ brains and aged microglia	RIPK3-dependent signaling enhances inflammasome activation, and mitochondrial ROS acts as a common upstream trigger of PCD	[191,192,193,194]
Ferroptosis	Iron accumulation, Lipid peroxidation, and antioxidant failure	GPX4	Reduced GPX4 expression in the AD mouse model and elevated lipid peroxidation in AD brains	Oxidative stress and mitochondrial dysfunction facilitate ferroptosis–pyroptosis–necroptosis coupling	[171,189,191,192,193,194]

Abbreviations: AD, Alzheimer’s disease; DAMPs, Damage-associated molecular patterns; GSDMD, Gasdermin D; Glutathione Peroxidase 4, GPX4; PCD, programmed cell death; ROS, reactive oxygen species.

## Data Availability

No new data were created or analyzed in this study.
